# Induction of Strong Magneto-Optical Effect and High Compatibility with Si of BiFeO_3_ Thin Film by Sr and Ti Co-Doping

**DOI:** 10.3390/ma18132953

**Published:** 2025-06-22

**Authors:** Nanxi Lin, Hong Zhang, Yunye Shi, Chenjun Xu, Zhuoqian Xie, Yunjin Chen

**Affiliations:** 1 Key Laboratory of Non-Destructive Testing Technology, Fujian Polytechnic Normal University, Fuqing 350300, China; 2 Yunnan Invensight Optoelectronics Technology Co., Ltd., Kunming 650506, China

**Keywords:** magneto-optical materials, perovskite, bismuth ferrite, silicon-based, magnetic circle dichroism

## Abstract

The poor magnetic and magneto-optical properties of BiFeO_3_, along with its significant lattice mismatch with silicon, have limited its application in silicon-based integrated magneto-optical devices. In this study, co-doping with Sr^2+^ and Ti^4+^ ions effectively transformed the trigonal structure of BiFeO_3_ into a cubic phase, thereby reducing the lattice mismatch with silicon to 2.8%. High-quality, highly oriented, silicon-based cubic Sr,Ti:BiFeO_3_ thin films were successfully fabricated using radio frequency magnetron sputtering. Due to the induced lattice distortion, the characteristic periodic spiral spin antiferromagnetic structure of BiFeO_3_ was suppressed, resulting in a significant enhancement of the saturation magnetization of cubic Bi_0.5_Sr_0.5_Fe_0.5_Ti_0.5_O_3_ (48.0 emu/cm^3^), compared to that of pristine BiFeO_3_ (5.0 emu/cm^3^). Furthermore, the incorporation of Sr^2+^ and Ti^4+^ ions eliminated the birefringence effect inherent in trigonal BiFeO_3_, thereby inducing a pronounced magneto-optical effect in the cubic Sr,Ti:BiFeO_3_ thin film. The magnetic circular dichroic ellipticity (*ψ*_F_) of Bi_0.5_Sr_0.5_Fe_0.5_Ti_0.5_O_3_ reached an impressive 2300 degrees/cm.

## 1. Introduction

With the rapid advancement of optical communication and information technology, the demands for large-scale data storage, analysis, and transmission are continuously increasing, imposing higher performance requirements on magneto-optical devices such as optical isolators, optical storage, and optical modulators, all of which rely on magneto-optical materials at their core [1,2,3]. In addition, silicon-based optoelectronic integration technology represents one of the most important and actively pursued research directions. Alongside this, electronic components are increasingly evolving toward miniaturization and monolithic integration. Consequently, the development of magneto-optical thin-film materials exhibiting superior magneto-optical performance, high stability, low transmission loss, and strong compatibility with silicon substrates has become an urgent requirement for the progress of modern communication technologies.

Previous studies have shown that doping an appropriate amount of Bi^3+^ ions into garnet-type rare-earth ferrite magneto-optical materials such as RIG (R_3_Fe_5_O_12_, where R denotes rare earth elements) can significantly enhance the magneto-optical effect of these crystals. This enhancement is primarily attributed to the hybridization between the excited 6p orbitals of Bi^3+^ and the 3d orbitals of Fe^3+^, as well as the superexchange correction effect that leads to strong mixing between crystal field states of different energies, thereby greatly improving the magneto-optical properties. For example, Bi:YIG [4,5], Bi:TbIG [6], and Bi:GdIG [7] all exhibit excellent magneto-optical performance and large Faraday rotation angles. However, a significant lattice mismatch exists between garnet ferrites and silicon (YIG: 12.376 Å; Si: 5.431 Å), which often results in the cracking of garnet thin films grown on silicon substrates. This mismatch increases light scattering and transmission loss, thus limiting their potential application in integrated devices [8].

In contrast to RIG, perovskite-type ferrites such as RFeO_3_ provide a much better lattice match with silicon substrates and offer several advantages, including high sensitivity, superior magneto-optical performance, rapid response, and a high Curie temperature (620–760 K) [9,10,11]. As a typical perovskite multiferroic material, BiFeO_3_ is considered one of the most promising candidates for spintronic devices, magnetoelectric sensors, and high-density ferroelectric memory applications [12,13,14,15,16]. Given that Bi^3+^ can significantly enhance the magneto-optical effect in ferrites, BiFeO_3_, with its high Bi^3+^ concentration, should theoretically exhibit excellent magneto-optical properties. However, limited research on the magneto-optical characteristics and device applications of BiFeO_3_ thin films and single crystals has been reported. This is largely because BiFeO_3_ crystallizes in a trigonal system with an R3c rhombohedral space group and exhibits a unique helical G-type antiferromagnetic structure, resulting in very weak macroscopic magnetism [17,18]. Moreover, the birefringence effect inherent in trigonal crystals severely diminishes the magneto-optical performance. Additionally, impurity phases such as Bi_25_FeO_40_ and Bi_2_Fe_4_O_9_ are easily formed during BiFeO_3_ thin-film fabrication [19,20,21], hindering epitaxial growth on silicon substrates and degrading film quality.

It has been reported that ion doping can effectively tune the structure of ABO_3_ perovskites. According to the tolerance factor relation, when tolerance factor t falls within the range of 0.9–1.0, the formation of a cubic phase without birefringence becomes possible [21,22,23]. Furthermore, B-site ion doping helps disrupt the helical G-type antiferromagnetic structure in BiFeO_3_, potentially resulting in enhanced macroscopic magnetism. The cubic-phase BiFeO_3_ also possesses lattice parameters close to those of silicon, ensuring minimal lattice mismatch. Therefore, highly oriented, high-performance cubic-phase BiFeO_3_ magneto-optical films are expected to be achievable through ion doping and silicon substrate induction.

In this study, the structure of trigonal BiFeO_3_ was successfully modified by co-doping with Sr^2+^ and Ti^4+^ ions. A series of Bi_1−x_Sr_x_Fe_1−x_Ti_x_O_3_ (x = 0, 0.2, and 0.5) thin films was fabricated on (100)-oriented Si and SrTiO_3_ (STO) wafers, as well as on SiO_2_ quartz glass substrates, via radio frequency magnetron sputtering. The effects of Sr^2+^ and Ti^4+^ co-doping on the structure, magnetic properties, magneto-optical performance, growth orientation, film-forming quality, and permeability of BiFeO_3_ thin films were systematically investigated.

## 2. Materials and Methods

Bi_1−x_Sr_x_Fe_1−x_Ti_x_O_3_ (x = 0, 0.2, and 0.5) polycrystalline targets were synthesized using the high-temperature solid-state reaction method. First, the starting materials (Bi_2_O_3_, Fe_2_O_3_, SrCO_3_, and TiO_2_, Shanghai Aladdin Biochemical Technology Co., Ltd., Shanghai, China) were precisely weighed according to the stoichiometric ratios, thoroughly mixed, and pre-sintered at 700 °C for 4 h in an air atmosphere. Subsequently, a PVA ((CH_2_CHOH)_n_) binder (Shanghai Aladdin Biochemical Technology Co., Ltd., Shanghai, China) was added to the pre-sintered powder at a ratio of 0.05 mL/g. After complete grinding, the mixture was pressed into pellets under a pressure of 30 MPa and sintered at 820 °C for 4 h to obtain high-density polycrystalline targets.

Thin films of BiFeO_3_, Bi_0.8_Sr_0.2_Fe_0.8_Ti_0.2_O_3_, and Bi_0.5_Sr_0.5_Fe_0.5_Ti_0.5_O_3_ were deposited on SiO_2_ quartz glass, <100>-oriented silicon, and SrTiO_3_ (STO) wafers (Hefei Crystal Technical Material Co., Ltd., Hefei, China) using the RF magnetron sputtering technique. The detailed sputtering parameters are listed in Table 1. As the as-deposited thin films were amorphous, post-deposition annealing was necessary. To minimize the influence of oxygen vacancy defects on the optical and magnetic properties, the films were annealed at 600 °C for 3 h in a sufficient oxygen atmosphere to promote crystallization. The heating and cooling rates were carefully controlled at 1 °C/min and 0.7 °C/min, respectively.

The crystal phases of the thin films were analyzed using an X-ray diffractometer (XRD) (D/max-3C, Rigaku, Akishima, Japan) with a Cu Kα radiation source (λ = 1.5406 Å) operating at 30 kV and 15 mA. The grazing-incidence X-ray diffraction (GIXRD) spectra of the thin films were measured using the MPA-U4 thin-film platform. The thickness and surface morphology of the films were examined by scanning electron microscopy (SEM) (SU8000, Hitachi, Chiyoda, Japan) at a magnification of 70,000×, an accelerating voltage of 10 kV, and a working distance of 8–9 mm. The valence states and surface chemical composition were analyzed using X-ray photoelectron spectroscopy (XPS) (Escalab 250Xi, Thermo Scientific, Waltham, MA, USA), while the surface topography was characterized by atomic force microscopy (AFM) (Dimension Icon, Bruker, Manning Park Billerica, MA, USA). The transmittance spectra of the thin films, covering the wavelength range of 200–3000 nm, were measured using a UV-Vis-IR spectrometer (PerkinElmer Lambda 900, Waltham, MA, USA). The magnetic properties were evaluated with a vibrating sample magnetometer (LakeShore-7407, Westerville, OH, USA) at room temperature. To investigate the magnetic circular dichroism (MCD) effect, measurements were conducted using a circular dichroism spectrometer (MOS-450, Bio-Logic, Seyssinet-Pariset, France), with a magnetic field of 2500 Oe applied parallel to the light propagation direction and perpendicular to the film surface.

## 3. Results and Discussion

### 3.1. Crystalline Phase and Structure

**Figure 1:**
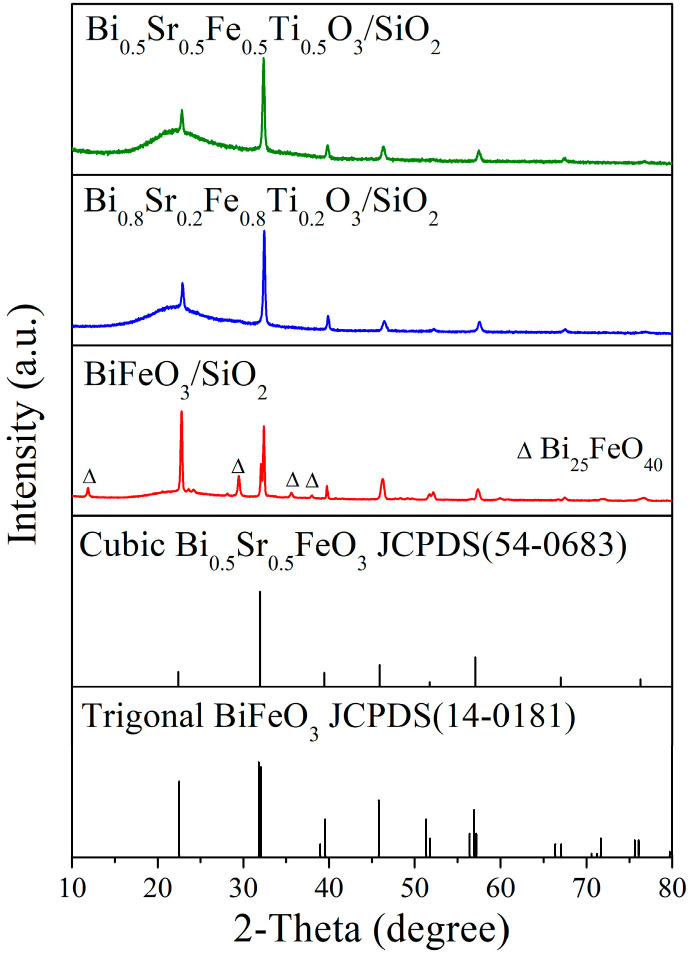
XRD patterns of Bi_1−x_Sr_x_Fe_1−x_Ti_x_O_3_/SiO_2_ thin films annealed at 600 °C.

According to the tolerance factor equation, the tolerance factor (t) of BiFeO_3_ was calculated to be 0.887. When the co-doping concentration of Sr^2+^ and Ti^4+^ ions exceeded 0.2, the t value increased above 0.9, suggesting the formation of a cubic phase BiFeO_3_. The XRD patterns of the annealed Bi_1−x_Sr_x_Fe_1−x_Ti_x_O_3_/SiO_2_ (x = 0, 0.2, and 0.5) thin films are presented in Figure 1. As shown in Figure 1, the undoped BiFeO_3_/SiO_2_ thin film exhibited a trigonal phase structure (JCPDS 54-0683) after annealing, along with the presence of a Bi_25_FeO_40_ impurity phase. In contrast, the Bi_0.8_Sr_0.2_Fe_0.8_Ti_0.2_O_3_/SiO_2_ thin film demonstrated good crystallinity without detectable Bi_25_FeO_40_ impurities. Notably, the two distinct peaks near 31.8° and 32° characteristic of the trigonal BiFeO_3_ phase merged into a single peak at 31.9° for the Bi_0.8_Sr_0.2_Fe_0.8_Ti_0.2_O_3_ thin film. All observed diffraction peaks could be attributed to the cubic phase (JCPDS 54-0683), indicating a phase transition from trigonal to cubic structure.

The XRD pattern of the Bi_0.5_Sr_0.5_Fe_0.5_Ti_0.5_O_3_/SiO_2_ thin film was similar to that of Bi_0.8_Sr_0.2_Fe_0.8_Ti_0.2_O_3_/SiO_2_; however, the diffraction peak near 51.7° was absent in the Bi_0.5_Sr_0.5_Fe_0.5_Ti_0.5_O_3_/SiO_2_ sample. This discrepancy may be attributed to compositional differences between the two films, resulting in slightly different crystallization behaviors under identical annealing conditions. Overall, the diffraction peaks observed for both Bi_0.5_Sr_0.5_Fe_0.5_Ti_0.5_O_3_/SiO_2_ and Bi_0.8_Sr_0.2_Fe_0.8_Ti_0.2_O_3_/SiO_2_ corresponded to the cubic phase of BiFeO_3_, confirming that co-doping with Sr^2+^ and Ti^4+^ ions induced a phase transformation from the trigonal to the cubic structure. Furthermore, the cubic-phase BiFeO_3_ appeared to possess enhanced phase stability, making it less susceptible to decomposition.

**Figure 2:**
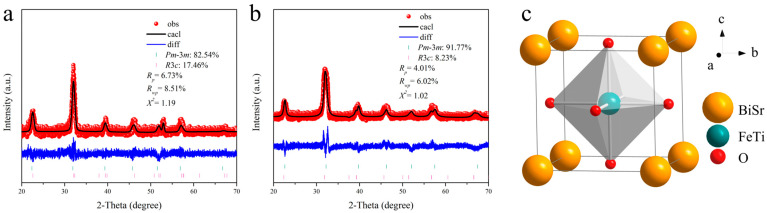
Fitted GIXRD spectra based on the Rietveld refinement of (**a**) Bi_0.8_Sr_0.2_Fe_0.8_Ti_0.2_O_3_/SiO_2_ and (**b**) Bi_0.5_Sr_0.5_Fe_0.5_Ti_0.5_O_3_/SiO_2_ thin films, and (**c**) unit cell structure diagram of cubic Bi_1−x_Sr_x_Fe_1−x_Ti_x_O_3_.

To determine the unit cell structure of cubic Sr,Ti:BiFeO_3_, the GIXRD patterns of Bi_0.8_Sr_0.2_Fe_0.8_Ti_0.2_O_3_/SiO_2_ and Bi_0.5_Sr_0.5_Fe_0.5_Ti_0.5_O_3_/SiO_2_ thin films were measured using a slow-scan mode with a scanning speed of 0.5°/min. Rietveld structure refinement was performed on the GIXRD patterns using cubic Bi_0.8_Sr_0.2_FeO_2.9_ and trigonal BiFeO_3_ crystal structure models, as shown in Figure 2a,b. The fitting spectra obtained from Rietveld refinement exhibited a high degree of agreement with the experimental diffraction patterns. The results revealed that the Bi_0.8_Sr_0.2_Fe_0.8_Ti_0.2_O_3_ thin film contained 82.54% of the cubic Pm-3m phase and 17.46% of the trigonal R3c phase. With increased doping content, the cubic Pm-3m phase fraction in Bi_0.5_Sr_0.5_Fe_0.5_Ti_0.5_O_3_ further rose to 91.77%. This enhancement was attributed to the higher Sr and Ti ion concentrations, which increased the tolerance factor (t) of Bi_1−x_Sr_x_Fe_1−x_Ti_x_O_3_, thereby promoting a higher cubic phase content [24]. Additionally, for the Bi_0.8_Sr_0.2_Fe_0.8_Ti_0.2_O_3_ thin film, the reliability factors, confidence factor (Rp), weighted profile R-factor (Rwp), and goodness-of-fit indicator (χ^2^), were 6.73%, 8.51%, and 1.19, respectively. For the Bi_0.5_Sr_0.5_Fe_0.5_Ti_0.5_O_3_ thin film, these values were 4.01%, 6.02%, and 1.02, respectively. These low error values suggest that the Rietveld refinement results were highly reliable for thin-film structural analysis. Table 2 presents the structural parameters obtained from Rietveld refinement, including the atomic positions and occupancy factors. The data show that Bi^3+^ and Sr^2+^ ions co-occupied the 1a site, Fe^3+^ and Ti^4+^ ions co-occupied the 1b site, and O^2−^ ions occupied the 3c site. Based on the Rietveld refinement results, the unit cell structure of cubic Bi_1−x_Sr_x_Fe_1−x_Ti_x_O_3_ was illustrated, as shown in Figure 2c.

**Figure 3:**
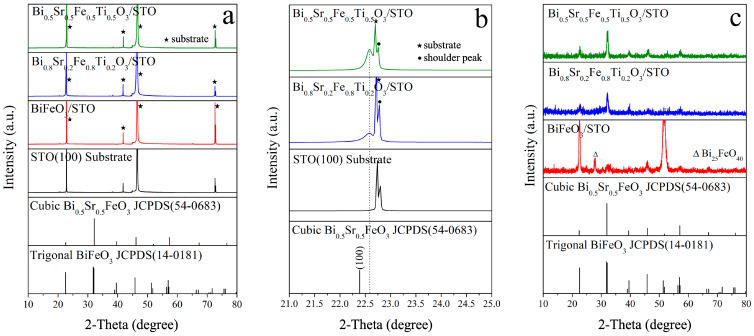
XRD patterns of (**a**) Bi_1−x_Sr_x_Fe_1−x_Ti_x_O_3_/STO thin films, (**b**) the cubic Bi_1−x_Sr_x_Fe_1−x_Ti_x_O_3_/STO thin films, and (**c**) the GIXRD patterns of Bi_1−x_Sr_x_Fe_1−x_Ti_x_O_3_/STO thin films.

Figure 3a presents the XRD patterns of the Bi_1−x_Sr_x_Fe_1−x_Ti_x_O_3_/STO thin films. Due to the strong diffraction effect of the single-crystal STO substrate, most of the thin-film diffraction peaks were obscured by the substrate peaks. However, the (100) diffraction peaks of the Bi_0.8_Sr_0.2_Fe_0.8_Ti_0.2_O_3_/STO and Bi_0.5_Sr_0.5_Fe_0.5_Ti_0.5_O_3_/STO thin films could be clearly observed at 22.6°, as shown in Figure 3b, indicating that the films deposited on the STO substrate exhibited a preferred (100) orientation. Compared to the standard cubic Bi_0.5_Sr_0.5_FeO_3_ reference card (JCPDS 54-0683), the (100) diffraction peak of the Bi_0.5_Sr_0.5_Fe_0.5_Ti_0.5_O_3_/STO film was shifted by 0.2° toward a higher angle. This shift was likely due to the lattice constant of the STO(001) substrate (a = 3.905 Å) being slightly smaller than that of the Sr,Ti:BiFeO_3_ film (a = 3.95 Å), which generated compressive strain in the in-plane direction of the film. This strain caused the film lattice to contract in-plane, reducing the lattice constant and resulting in a shift of the XRD peaks to higher angles [25]. Simultaneously, according to the Poisson effect [26], the out-of-plane lattice constant tended to elongate to compensate for this in-plane compression, leading to the formation of a tetragonal phase.

To minimize substrate interference and obtain more detailed information on the thin-film diffraction peaks, GIXRD patterns of the Bi_1−x_Sr_x_Fe_1−x_Ti_x_O_3_/STO thin films were also recorded, as shown in Figure 3c. It can be seen that all the diffraction peaks in the GIXRD patterns of the Bi_0.8_Sr_0.2_Fe_0.8_Ti_0.2_O_3_/STO and Bi_0.5_Sr_0.5_Fe_0.5_Ti_0.5_O_3_/STO thin films corresponded to the perovskite cubic phase. In contrast, most of the diffraction peaks of the BiFeO_3_/STO thin film corresponded to the perovskite trigonal phase, with fewer impurity phases compared to the BiFeO_3_/SiO_2_ film. This may be attributed to the fact that the SrTiO_3_ single-crystal substrate also possessed a perovskite structure, which may have stabilized the perovskite phase of BiFeO_3_ to some extent.

Furthermore, as shown in Figure 3a,b, the XRD and GIXRD diffraction peak intensities of the Bi_0.5_Sr_0.5_Fe_0.5_Ti_0.5_O_3_/STO thin film were stronger than those of the Bi_0.8_Sr_0.2_Fe_0.8_Ti_0.2_O_3_/STO thin film, indicating a higher degree of crystallization. This result differed from that in Figure 1 and may have been due to the higher Sr^2+^ and Ti^4+^ ion doping concentration in Bi_0.5_Sr_0.5_Fe_0.5_Ti_0.5_O_3_, which improved lattice compatibility with the STO substrate and promoted thin-film growth.

**Figure 4:**
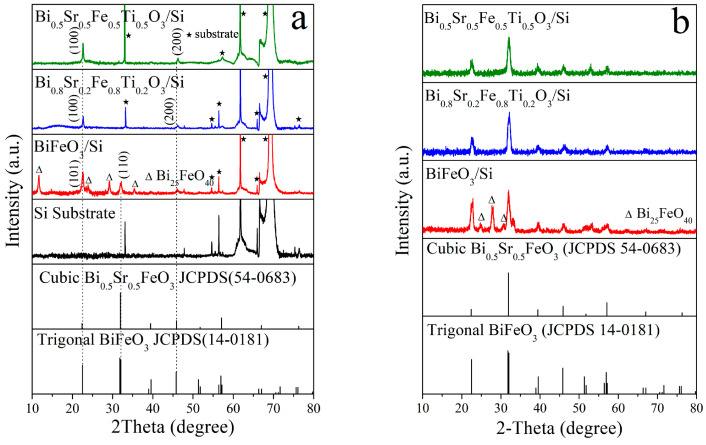
(**a**) XRD and (**b**) GIXRD patterns of Bi_1−x_Sr_x_Fe_1−x_Ti_x_O_3_/Si thin films.

**Figure 5:**
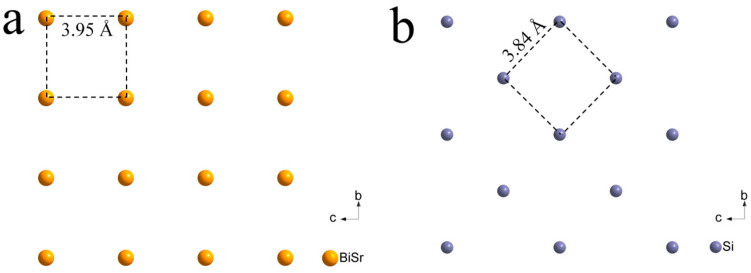
Lattice diagram of (**a**) cubic Bi_1−x_Sr_x_Fe_1−x_Ti_x_O_3_(100) and (**b**) Si(100).

The XRD and GIXRD patterns of Bi_1−x_Sr_x_Fe_1−x_Ti_x_O_3_/Si thin films are shown in Figure 4a,b. According to the XRD patterns, apart from the diffraction peaks from the silicon substrate, only the (100) and (200) plane diffraction peaks of the Bi_0.8_Sr_0.2_Fe_0.8_Ti_0.2_O_3_/Si and Bi_0.5_Sr_0.5_Fe_0.5_Ti_0.5_O_3_/Si thin films were observed. This indicated that the cubic phase Bi_1−x_Sr_x_Fe_1−x_Ti_x_O_3_ films grown on Si(100) substrates exhibited a distinct (100) plane preferred orientation. This preferred orientation was primarily due to the low lattice mismatch between the cubic Sr,Ti:BiFeO_3_ thin films and the silicon substrate. Figure 5 illustrates the lattice structure of cubic Sr,Ti:BiFeO_3_(100) and Si(100). Based on the lattice mismatch formula, the mismatch between Sr,Ti:BiFeO_3_(100) and Si(100) was calculated to be only 2.8%. The GIXRD patterns of Bi_1−x_Sr_x_Fe_1−x_Ti_x_O_3_/Si thin films provided additional diffraction information. All diffraction peaks for the Bi_0.8_Sr_0.2_Fe_0.8_Ti_0.2_O_3_/Si and Bi_0.5_Sr_0.5_Fe_0.5_Ti_0.5_O_3_/Si thin films corresponded to the perovskite cubic phase. Overall, while the cubic phase Bi_1-x_Sr_x_Fe_1-x_Ti_x_O_3_/Si thin films remained polycrystalline, they exhibited a clear (100) plane preferred orientation. In contrast, the BiFeO_3_/Si thin film showed diffraction peaks corresponding to the (101) and (110) planes of the trigonal phase in the XRD pattern (Figure 4a). Moreover, additional peaks matching the standard reference card (JCPDS 14-0181) were visible in the GIXRD pattern (Figure 4b), confirming that the film retained a trigonal crystal structure with a minor presence of the secondary Bi_25_FeO_40_ phase.

In summary, Sr^2+^ and Ti^4+^ ion co-doping effectively modulated the crystal structure of BiFeO_3_ from the trigonal to the cubic phase, significantly improving lattice matching with silicon. This represents a promising strategy for the fabrication of high-quality, silicon-based cubic Sr,Ti:BiFeO_3_ thin films suitable for integration into silicon-based devices.

### 3.2. Surface Morphology and Thickness

**Figure 6:**
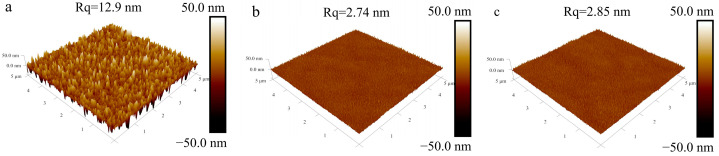
AFM 3D images of (**a**) annealed BiFeO_3_/Si, (**b**) Bi_0.8_Sr_0.2_Fe_0.8_Ti_0.2_O_3_/Si, and (**c**) Bi_0.5_Sr_0.5_Fe_0.5_Ti_0.5_O_3_/Si thin films.

The film-forming quality of the samples was evaluated using AFM, as shown in Figure 6. The AFM images revealed that the surfaces of the Bi_1−x_Sr_x_Fe_1−x_Ti_x_O_3_/Si films exhibited well-developed crystallization with uniformly distributed particles and no visible cracks. The root mean square (RMS) roughness (Rq) values for the Bi_0.8_Sr_0.2_Fe_0.8_Ti_0.2_O_3_/Si and Bi_0.5_Sr_0.5_Fe_0.5_Ti_0.5_O_3_/Si films were measured to be 2.74 nm and 2.85 nm, respectively, i.e., significantly lower than the 12.9 nm roughness of the BiFeO_3_/Si thin film prepared under identical conditions.

**Figure 7:**
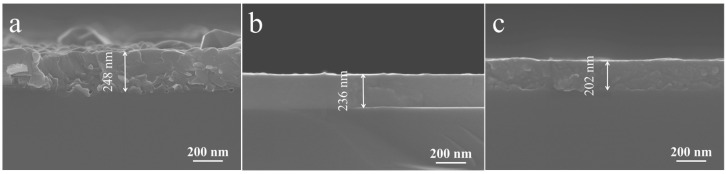
Cross-section SEM images of annealed (**a**) BiFeO_3_/Si, (**b**) Bi_0.8_Sr_0.2_Fe_0.8_Ti_0.2_O_3_/Si, and (**c**) Bi_0.5_Sr_0.5_Fe_0.5_Ti_0.5_O_3_/Si thin films.

The surface morphology and thickness of the films were analyzed using SEM, as shown in Figure 7. The measured thicknesses of the BiFeO_3_/Si, Bi_0.8_Sr_0.2_Fe_0.8_Ti_0.2_O_3_/Si, and Bi_0.5_Sr_0.5_Fe_0.5_Ti_0.5_O_3_/Si thin films after 1.5 h of sputtering were 248 nm, 236 nm, and 202 nm, respectively. The SEM images also revealed that the BiFeO_3_/Si thin film exhibited a rough surface with uneven grain sizes, which could be attributed to the large lattice mismatch between BiFeO_3_ and the silicon substrate. This mismatch led to strong optical scattering at the grain boundaries and negatively affected the optical transmission properties of the thin film. In contrast, the Bi_0.8_Sr_0.2_Fe_0.8_Ti_0.2_O_3_/Si and Bi_0.5_Sr_0.5_Fe_0.5_Ti_0.5_O_3_/Si thin films exhibited significantly improved film-forming quality, with fewer grain boundaries and smoother surfaces. This enhancement was due to the reduced lattice mismatch between the cubic-phase Sr,Ti:BiFeO_3_ and the silicon substrate, which strengthened the film-substrate adhesion and promoted superior film formation.

### 3.3. Valence State

**Figure 8:**
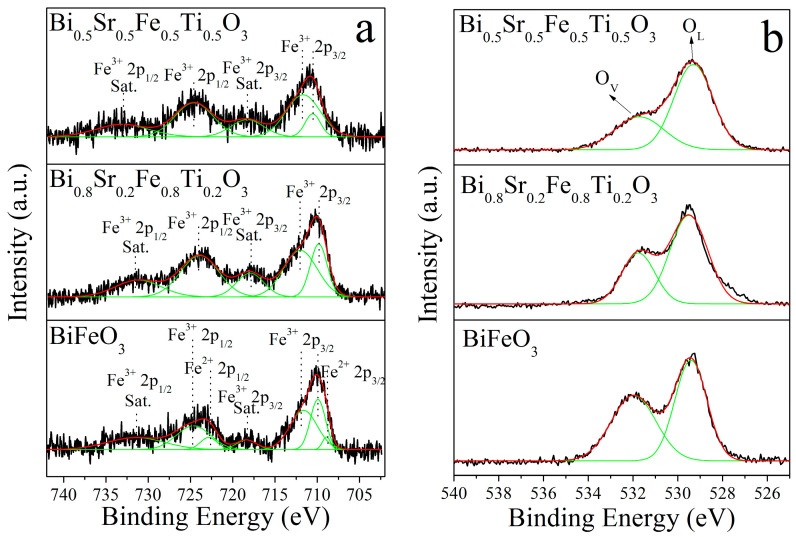
(**a**) Fe 2p, (**b**) O 1s XPS spectra and fitting curves of Bi_1−x_Sr_x_Fe_1−x_Ti_x_O_3_/Si thin films.

The presence of Fe^2+^ ions and oxygen vacancies can influence the optical transmittance of a film, while the valence state of Fe ions also affects its magnetic properties. Figure 8a shows the Fe 2p XPS spectra of Bi_1−x_Sr_x_Fe_1−x_Ti_x_O_3_/Si thin films. According to the relevant literature, the Fe^3+^ 2p_1/2_ characteristic peak and its satellite peak were located at 724 eV and 733 eV, respectively [27]. The Fe^3+^ 2p_3_/_2_ characteristic peak appeared within the range of 710–713 eV, with its corresponding satellite peak at 718 eV [27,28]. Thus, the Fe ions in the cubic Bi_0.8_Sr_0.2_Fe_0.8_Ti_0.2_O_3_/Si and Bi_0.5_Sr_0.5_Fe_0.5_Ti_0.5_O_3_/Si thin films were confirmed to be entirely in the trivalent state. In contrast, the Fe 2p XPS spectrum of the BiFeO_3_/Si thin film displayed not only the Fe^3+^ peaks but also characteristic peaks corresponding to Fe^2+^ 2p_1/2_ and Fe^2+^ 2p_3/2_ states, indicating the partial presence of Fe^2+^ ions. The fitting peak area ratio (Fe^2+^/(Fe^3+^ + Fe^2+^)) of the lowest binding energy states was used to estimate the relative Fe^2+^ content in the BiFeO_3_/Si film, calculated to be approximately 5.3%. This may have been due to the tendency of BiFeO_3_ films to form the Bi_25_FeO_40_ impurity phase during preparation, leading to the partial reduction of Fe^3+^ to Fe^2+^ ions. The O 1s XPS spectra of Bi_1−x_Sr_x_Fe_1−x_Ti_x_O_3_/Si thin films are shown in Figure 8b. According to the literature, the peak at 529.7 eV (O_L_) corresponded to lattice oxygen, while the peak at 531.7 eV (O_V_) was associated with low-valence oxygen species and oxygen vacancy defects [29,30]. As observed in the spectra, all films, including BiFeO_3_/Si, Bi_0.8_Sr_0.2_Fe_0.8_Ti_0.2_O_3_/Si, and Bi_0.5_Sr_0.5_Fe_0.5_Ti_0.5_O_3_/Si, exhibited both lattice oxygen and oxygen vacancy-related peaks. However, with increasing Sr^2+^ and Ti^4+^ co-doping content, the intensity of the 531.7 eV peak decreased significantly. This indicated that co-doping with Sr^2+^ and Ti^4+^ effectively reduced the concentration of low-valence oxygen states and oxygen vacancy defects in BiFeO_3_.

### 3.4. Transmittance

**Figure 9:**
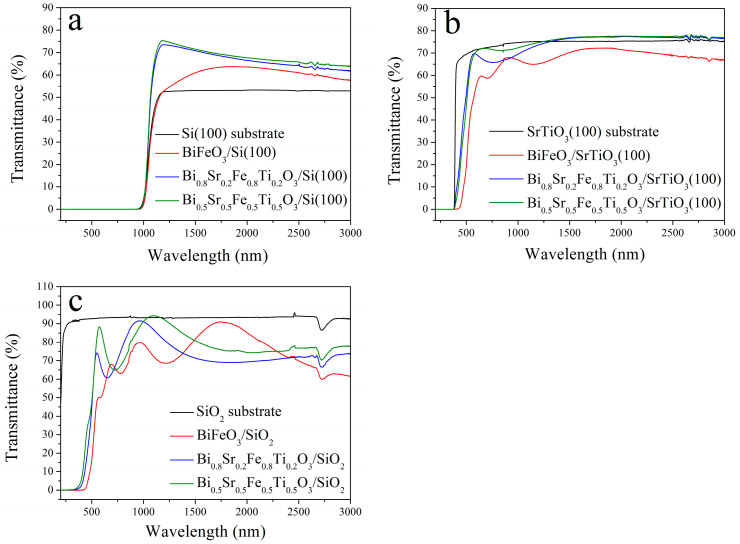
Transmittance spectra of the Bi_1−x_Sr_x_Fe_1−x_Ti_x_O_3_ thin films deposited on (**a**) Si, (**b**) STO and (**c**) Si substrates.

Studying the optical loss mechanisms of thin films is essential for improving the magneto-optical performance of materials and for developing low-loss, silicon-based optical isolation devices. Figure 9a shows the transmittance spectra of annealed Bi_1−x_Sr_x_Fe_1−x_Ti_x_O_3_/Si thin films in the UV-VIS and near-infrared regions. As shown, the transmittance curves of Bi_0.8_Sr_0.2_Fe_0.8_Ti_0.2_O_3_/Si (236 nm) and Bi_0.5_Sr_0.5_Fe_0.5_Ti_0.5_O_3_/Si (202 nm) were very similar, indicating that film thickness had little influence on the transmittance. In contrast, the lower transmittance of the BiFeO_3_/Si film could be mainly attributed to its greater surface roughness, as evidenced in Figure 6a and Figure 7a, which led to increased optical scattering and higher transmission losses.

The Bi_0.8_Sr_0.2_Fe_0.8_Ti_0.2_O_3_/Si and Bi_0.5_Sr_0.5_Fe_0.5_Ti_0.5_O_3_/Si films exhibited transmittance values ranging from 60% to 75% in the 1000–3000 nm wavelength range, i.e., significantly higher than that of the silicon substrate. This improvement was mainly due to the large absorption of the bare silicon wafer; when coated with high-quality thin films, an anti-reflection effect occurred, enhancing transmittance. Moreover, the transmittance curves of the Bi_0.8_Sr_0.2_Fe_0.8_Ti_0.2_O_3_/Si and Bi_0.5_Sr_0.5_Fe_0.5_Ti_0.5_O_3_/Si films were smooth, owing to the similar refractive indices of the Si substrate and the perovskite ferrite films, which weakened the light interference effect between them. Additionally, the absence of discernible absorption peaks in the transmittance spectra of the cubic Bi_1−x_Sr_x_Fe_1−x_Ti_x_O_3_/Si films suggested high film quality and low optical loss, rendering these films suitable for integration into silicon-based magneto-optical devices.

In contrast, the trigonal BiFeO_3_/Si film showed significantly lower transmittance compared to the cubic Bi_1−x_Sr_x_Fe_1−x_Ti_x_O_3_/Si films. This could be attributed to its higher surface roughness, stronger grain boundary scattering, and higher concentration of oxygen vacancies, all of which contributed to greater optical loss and reduced transmission performance. Furthermore, the transmittance curve of the BiFeO_3_/Si film showed a pronounced decline below 1800 nm, likely due to the presence of Fe^2+^ ions that exhibited absorption near 1200 nm [31].

Overall, the superior film quality and improved lattice matching between the cubic Bi_1−x_Sr_x_Fe_1−x_Ti_x_O_3_ films and the silicon substrate resulted in excellent optical properties and much higher transmittance compared to the BiFeO_3_/Si film. Similarly, as shown in Figure 9b, the Bi_0.8_Sr_0.2_Fe_0.8_Ti_0.2_O_3_/STO and Bi_0.5_Sr_0.5_Fe_0.5_Ti_0.5_O_3_/STO thin films also exhibited good transmittance, ranging from 70% to 80% in the visible and near-infrared regions. However, due to the presence of minor impurity phases, the transmittance of the BiFeO_3_/STO film was reduced to approximately 60–70%.

Since the absorption edges of Si and STO were located near 1000 nm and 380 nm, respectively, the absorption edges of the Bi_1-x_Sr_x_Fe_1-x_Ti_x_O_3_/Si and Bi_1−x_Sr_x_Fe_1−x_Ti_x_O_3_/STO films were influenced by those of the respective substrates. To obtain the intrinsic absorption edge of the Bi_1−x_Sr_x_Fe_1−x_Ti_x_O_3_ films, their transmittance spectra were also measured on SiO_2_ substrates, as shown in Figure 9c. The transmittance curves displayed periodic oscillations caused by optical interference effects resulting from the refractive index differences between the substrate and the film [32].

Moreover, Sr^2+^ and Ti^4+^ co-doping caused a significant blue shift in the absorption edge of the cubic Bi_1−x_Sr_x_Fe_1−x_Ti_x_O_3_ thin films compared to the trigonal BiFeO_3_ film. The absorption edges for BiFeO_3_, Bi_0.8_Sr_0.2_Fe_0.8_Ti_0.2_O_3_, and Bi_0.5_Sr_0.5_Fe_0.5_Ti_0.5_O_3_ films were 423 nm, 319 nm, and 312 nm, respectively. This shift was primarily attributed to the smaller grain sizes of the cubic-phase Bi_1−x_Sr_x_Fe_1−x_Ti_x_O_3_ films compared to the trigonal-phase BiFeO_3_ film. The resulting quantum confinement effect increased the energy level separation as the grain size decreased, causing a blue shift in the absorption edge [33].

### 3.5. Magnetism

**Figure 10:**
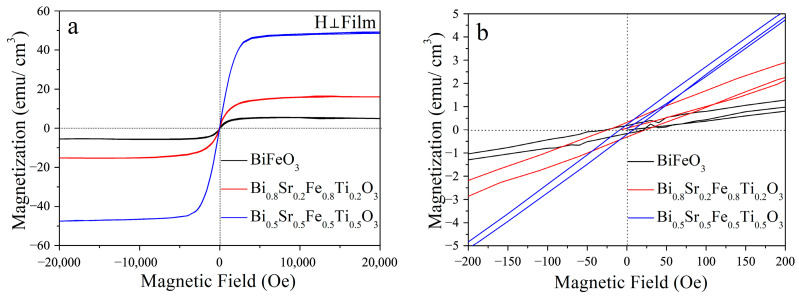
(**a**) Magnetic hysteresis loop and (**b**) coercive force of Bi_1−x_Sr_x_Fe_1−x_Ti_x_O_3_/Si thin films.

Figure 10 shows the magnetic hysteresis loops of Bi_1−x_Sr_x_Fe_1−x_Ti_x_O_3_/Si thin films measured at room temperature. The maximum applied magnetic field was 20,000 Oe, with the field direction oriented perpendicular to the film surface (out-of-plane). As seen in Figure 10a, the saturation magnetization (M_s_) of the Bi_1−x_Sr_x_Fe_1−x_Ti_x_O_3_/Si films significantly increased with the Sr^2+^ and Ti^4+^ co-doping concentration. Specifically, the M_s_ of the BiFeO_3_/Si film was only 5.0 emu/cm^3^, whereas that of the Bi_0.8_Sr_0.2_Fe_0.8_Ti_0.2_O_3_/Si and Bi_0.5_Sr_0.5_Fe_0.5_Ti_0.5_O_3_/Si films rose to 17.5 emu/cm^3^ and 48.0 emu/cm^3^, respectively. Compared to trigonal BiFeO_3_, the cubic Bi_0.5_Sr_0.5_Fe_0.5_Ti_0.5_O_3_ film exhibited a markedly enhanced magnetic response. This improvement was primarily attributed to the substitution of Fe^3+^ ions by Ti^4+^ ions at the B-site, which disrupted the helical G-type antiferromagnetic structure inherent to BiFeO_3_, thereby significantly enhancing its macroscopic magnetization [34]. Additionally, as shown in Figure 10b, the coercive force (*H*_c_) values for the BiFeO_3_/Si and Bi_0.8_Sr_0.2_Fe_0.8_Ti_0.2_O_3_/Si thin films were both approximately 50 Oe. In contrast, the Hc of the Bi_0.5_Sr_0.5_Fe_0.5_Ti_0.5_O_3_/Si film decreased to 20 Oe, indicating improved soft magnetic properties. According to previous studies, magneto-optical materials that exhibit high saturation magnetization and low coercivity are well-suited for use in magneto-optical modulators, switches, sensors, and related devices [35,36]. Therefore, the Bi_0.5_Sr_0.5_Fe_0.5_Ti_0.5_O_3_/Si thin film, with its combination of high saturation magnetization and low coercivity, holds great promise for application in these magneto-optical technologies.

### 3.6. Magneto-Optical Performance

**Figure 11:**
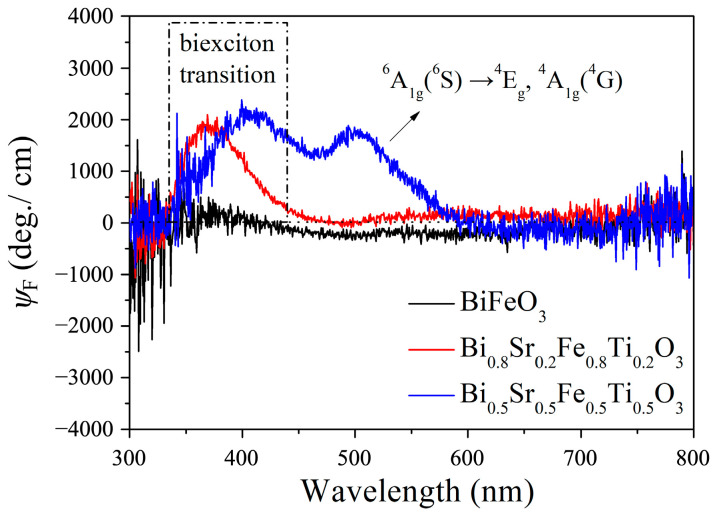
MCD spectra of Bi_1−x_Sr_x_Fe_1−x_Ti_x_O_3_/SiO_2_ thin films.

The MCD arises from the difference in absorption coefficients between left-handed and right-handed circularly polarized light in the presence of a magnetic field. Materials exhibiting a strong MCD response have promising applications in optical modulation, sensing, spintronics, magneto-optical imaging, and related fields. Figure 11 presents the MCD spectra of Bi_1−x_Sr_x_Fe_1−x_Ti_x_O_3_/SiO_2_ thin films in the 300–800 nm wavelength range, measured under an applied magnetic field of 2500 Oe. As shown, the BiFeO_3_/SiO_2_ film exhibited no detectable MCD signal under these conditions, whereas the Bi_0.8_Sr_0.2_Fe_0.8_Ti_0.2_O_3_/SiO_2_ and Bi_0.5_Sr_0.5_Fe_0.5_Ti_0.5_O_3_/SiO_2_ thin films displayed distinct MCD signal peaks, indicating a pronounced magnetic circular dichroism effect. For the Bi_0.8_Sr_0.2_Fe_0.8_Ti_0.2_O_3_/SiO_2_ film, a strong MCD peak appeared at 372 nm, attributed to the paramagnetic double exciton transition, with a corresponding MCD ellipticity (*ψ*_F_) value of approximately 2000 deg./cm. In contrast, the Bi_0.5_Sr_0.5_Fe_0.5_Ti_0.5_O_3_/SiO_2_ film exhibited prominent MCD peaks at around 400 nm and 500 nm, corresponding to a double exciton transition and ^6^A_1g_(^6^S)→^4^E_9_, ^4^A_1g_(^4^G) electronic transitions, respectively [37,38]. The maximum MCD *ψ*_F_ value reached 2200 deg./cm, exceeding that of Ce^3+^:YIG (1700 deg./cm under a magnetic field of 5000 Oe) [39]. This result indicated that the magneto-optical effect of Bi_1−x_Sr_x_Fe_1−x_Ti_x_O_3_ was significantly enhanced with increasing Sr^2+^ and Ti^4+^ co-doping levels. Notably, the MCD signal corresponding to the ^6^A_1g_(^6^S)→^4^E_9_, ^4^A_1g_(^4^G) transition appeared in the Bi_0.5_Sr_0.5_Fe_0.5_Ti_0.5_O_3_ film but was absent in the Bi_0.8_Sr_0.2_Fe_0.8_Ti_0.2_O_3_ film. This may have been due to the comparatively weaker magnetism of Bi_0.8_Sr_0.2_Fe_0.8_Ti_0.2_O_3_, which rendered this transition undetectable in its MCD spectrum.

**Figure 12:**
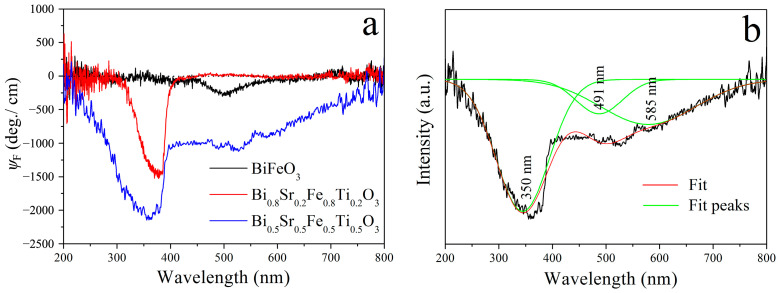
(**a**) MCD spectra and (**b**) fitting curves of Bi_1−x_Sr_x_Fe_1−x_Ti_x_O_3_/STO thin films.

In addition, the MCD spectra of Bi_1−x_Sr_x_Fe_1−x_Ti_x_O_3_/STO films in the 200–800 nm wavelength range were also measured under an external magnetic field of 2500 Oe, as shown in Figure 12. It can be seen that the BiFeO_3_/STO film exhibited a weak MCD signal at around 500 nm, with a corresponding MCD ellipticity (*ψ*_F_) value of only 200 deg./cm. The MCD peak positions of the Bi_0.8_Sr_0.2_Fe_0.8_Ti_0.2_O_3_/STO film were similar to those observed for the Bi_0.8_Sr_0.2_Fe_0.8_Ti_0.2_O_3_/SiO_2_ film; however, its maximum |*ψ*_F_| value decreased to 1490 deg./cm. This reduction may be attributed to differences in substrate properties, such as crystallographic orientation, thickness, absorption characteristics, and optical interference effects. Gaussian fitting of the MCD spectrum of the Bi_0.5_Sr_0.5_Fe_0.5_Ti_0.5_O_3_/STO film was performed, as shown in Figure 12b. The fitted spectrum revealed three distinct peaks located at 350 nm, 491 nm, and 585 nm, corresponding to a double exciton transition, a ^6^A_1g_(^6^S)→^4^E_9_, ^4^A_1g_(^4^G) transition, and a ^6^A_1g_(^6^S)→^4^T_1g_(^4^G) transition, respectively [36,37]. The maximum |*ψ*_F_| value reached 2300 deg./cm, comparable to that of the Bi_0.5_Sr_0.5_Fe_0.5_Ti_0.5_O_3_/SiO_2_ thin film. Overall, Sr^2+^ and Ti^4+^ co-doping effectively suppressed the detrimental birefringence effects of trigonal BiFeO_3_ while significantly enhancing the magnetic properties of the BiFeO_3_ films. As a result, the magneto-optical performance of the Bi_1−x_Sr_x_Fe_1−x_Ti_x_O_3_ thin films was markedly improved. Notably, compared with the widely used magneto-optical material Ce^3+^:YIG, the Bi_0.5_Sr_0.5_Fe_0.5_Ti_0.5_O_3_ thin film demonstrates a stronger magneto-optical effect under a much lower applied magnetic field.

## 4. Conclusions

In this study, the tolerance factor (t) of BiFeO_3_ was effectively modulated by co-doping with Sr^2+^ and Ti^4+^ ions, resulting in a structural transformation from the trigonal to the cubic phase. Rietveld refinement analysis revealed that as the doping concentration (x) increased from 0.2 to 0.5, the fraction of the Pm-3m cubic phase in Bi_1−x_Sr_x_Fe_1−x_Ti_x_O_3_ thin films increased from 82.54% to 91.77%, thereby significantly reducing the lattice mismatch with the silicon substrate. High-quality, <100>-oriented cubic Sr,Ti:BiFeO_3_ thin films were successfully fabricated on Si substrates via RF magnetron sputtering. In contrast, Sr,Ti:BiFeO_3_ films deposited on STO substrates exhibited a tetragonal structure due to the presence of compressive strain. Furthermore, Sr^2+^ and Ti^4+^ co-doping reduced the concentration of oxygen vacancies and Fe^2+^ ions, which improved the optical transmittance properties of the Bi_1−x_Sr_x_Fe_1−x_Ti_x_O_3_ films. With increasing Sr^2+^ and Ti^4+^ doping levels, the magnetic properties of the Sr,Ti:BiFeO_3_ films were markedly enhanced. The highest saturation magnetization, 48.0 emu/cm^3^, was observed for the Bi_0.5_Sr_0.5_Fe_0.5_Ti_0.5_O_3_ thin film, significantly exceeding that of BiFeO_3_ (5.0 emu/cm^3^) and Bi_0.8_Sr_0.2_Fe_0.8_Ti_0.2_O_3_ (17.5 emu/cm^3^). In addition to superior magnetic properties, the Sr,Ti:BiFeO_3_ thin films exhibited excellent magneto-optical performance. The maximum MCD *ψ*_F_ value of the Bi_0.5_Sr_0.5_Fe_0.5_Ti_0.5_O_3_ thin film reached 2300 deg./cm, surpassing that of widely used Ce^3+^:YIG magneto-optical films. Therefore, cubic-phase Sr,Ti:BiFeO_3_ thin films, with their strong magnetic and magneto-optical effects and high compatibility with silicon substrates, show great potential for application in integrated magneto-optical devices such as modulators, sensors, switches, and silicon-based monolithic integrated magneto-optical isolators.

## Figures and Tables

**Table 1:** Radio frequency magnetron sputtering parameters for depositing thin films.

Catalog	Parameter
Target	Bi_1−x_Sr_x_Fe_1−x_Ti_x_O_3_ (x = 0, 0.2, and 0.5)
Substrate	SiO_2_ quartz glass, Si(100) and STO(100)
Substrate temperature	room temperature
Substrate-target distance (cm)	5.0
Background pressure (Pa)	1 × 10^−4^
Sputter gas	Ar
Sputter gas flow (Sccm)	20
Sputter pressure (Pa)	1.6
Radio frequency power (W)	80
Deposition time (min)	90

**Table 2:** The Rietveld refinement data of a cubic Bi_1−x_Sr_x_Fe_1−x_Ti_x_O_3_/SiO_2_ thin film.

Chemical Formula	Atom	Wyckoff-Site	*x*	*y*	*z*	Occupancy
Bi_0.8_Sr_0.2_Fe_0.8_Ti_0.2_O_3_	Bi_1_	1a	0	0	0	0.8
	Sr_1_	1a	0	0	0	0.2
	Fe_1_	1b	0.5	0.5	0.5	0.8
	Ti_1_	1b	0.5	0.5	0.5	0.2
	O_1_	3c	0	0.5	0.5	1
Bi_0.5_Sr_0.5_Fe_0.5_Ti_0.5_O_3_	Bi_1_	1a	0	0	0	0.5
	Sr_1_	1a	0	0	0	0.5
	Fe_1_	1b	0.5	0.5	0.5	0.5
	Ti_1_	1b	0.5	0.5	0.5	0.5
	O_1_	3c	0	0.5	0.5	1

## Data Availability

The original contributions presented in the study are included in the article; further inquiries can be directed to the corresponding author.
